# Clinicopathological Challenges in Tumors of the Nasal Cavity and Paranasal Sinuses: Our Experience

**DOI:** 10.7759/cureus.29128

**Published:** 2022-09-13

**Authors:** Subhra Kumari, Surabhi Pandey, Mamta Verma, Amit Kumar Rana, Swati Kumari

**Affiliations:** 1 Pathology, Shri Ram Murti Smarak Institute of Medical Sciences, Bareilly, IND; 2 Otorhinolaryngology, Shri Ram Murti Smarak Institute of Medical Sciences, Bareilly, IND

**Keywords:** angiomatous nasal polyp (anp), hpe, polyps, nasal tumours, angiofibroma, schneiderian, scc, mucormycosis, inverted papilloma

## Abstract

Background and objective

Nasal and paranasal lesions are one of the most common otorhinolaryngological presentations encountered in clinical practice. Common presenting symptoms of these lesion range from nasal blockades, facial swellings, pain, nasal discharge, and epistaxis to orbital and ear symptoms. Diagnosis can be tricky as these symptoms are common in inflammatory conditions and tumors. The aim of our study was to observe the epidemiology and clinical pathological findings in patients with nasal and paranasal masses presenting to our institute and discuss the challenges in proper diagnosis and management due to similar presentations, and the role of histopathological examination (HPE) and immunohistochemistry (IHC) in overcoming these challenges.

Methods

The IPD records of 396 patients were taken up for the study. All the specimens were sent in 10% neutral buffered formalin for examination as biopsy for diagnosis or after surgical excision. After adequate fixation, the biopsy specimen was submitted for routine processing, followed by paraffin embedding, and stained with hematoxylin and eosin (H&E). Special stains like periodic acid-Schiff (PAS) and Ziehl-Neelsen (ZN) stains for acid-fast bacilli (AFB) were used as required. IHC was performed in the required samples. IHC markers were performed on representative paraffin-embedded sections according to the streptavidin-biotin immunoperoxidase technique as needed. The findings were noted, and histology was correlated with clinical presentations and investigations, tabulated, and statistically analyzed using SPSS Statistics (IBM, Armonk, NY).

Results

Of note, 67.92%% were non-neoplastic lesions whereas 18.18% came out to be benign neoplasms and 13.88% were malignant lesions on HPE. Nasal obstruction was the most common presenting symptom (73.23% of patients) followed by nasal mass (64.14% of cases). Inflammatory sinonasal polyps were the most prominent cases in our study, accounting for 41.16% of all lesions; 18.68% fungal RS (mucormycosis) were seen in our study and 4.54% were cases of juvenile nasopharyngeal angiofibroma (JNA). The majority of benign neoplasms encountered were Schneiderian papilloma or inverted papilloma (06.81%). Eighteen (4.54%) cases of squamous cell carcinomas (SCC) were seen in our study and 2.77% (n=11) cases were of adenoid cystic carcinoma. Of 18 cases of SCC, moderately differentiated SCC carcinoma accounted for 10 cases followed by poorly differentiated SCC (5/18) and nonkeratinizing SCC (3/18). IHC for p40 was performed in all the cases of nonkeratinizing SCC, which showed strong and diffuse nuclear positivity.

Conclusion

The nasal cavity is the site of the most varied presentation of tumors in the upper respiratory tract. Mass in the nose and paranasal sinus (PNS) form a heterogeneous group of lesions with varied histopathological features. The proximity of the area to the eyes and brain warrants early definitive diagnosis so that the lesion is treated before it can involve important and vital centers. Even though malignant nasal tumors have a very low incidence, they cause a lot of morbidity due to their long course and frequent local recurrences. Nasal tumors tend to become polypoidal. Epithelioid papilloma of the nasal cavity often resembles a nasal polyp. Clinical diagnosis can be challenging due to similar presentations and appearances, and hence histological examination is a vital tool for the timely diagnosis of such patients.

## Introduction

Nasal and paranasal lesions are otorhinolaryngological presentations frequently encountered in clinical practice for diagnosis and management. A variety of non-neoplastic and neoplastic pathologies are seen in routine practice. Sinonasal cavities present with a histopathologically diverse group of masses in a small, complex area. Polypoidal masses are the most common lesions encountered in the nasal cavity. Malignant lesions of the sinonasal tract account for approximately 3% of head-neck malignancies [1]. Both epithelial and non-epithelial neoplasms have been reported in the nose and paranasal sinuses. Common presenting symptoms of these lesion range from nasal blockades, facial swellings, pain, nasal discharge, and epistaxis to orbital and ear symptoms [2]. Diagnosis is tricky because these symptoms are common in inflammatory conditions and tumors.

The sinonasal tract is a peculiar area as almost every type of tumor can form in this area. Most clinicians make a presumptive diagnosis based on symptoms, clinical findings, and advanced radiological techniques. Still, histopathological examination (HPE) remains the gold standard for diagnosis. Accurate HPE can make a lot of difference in management direction and follow-up of certain conditions, especially in rare conditions that have a similar appearance to common benign conditions [3]. Also, there are pathological conditions that are benign to start with but may transition to locally invasive or malignant forms if not managed at an earlier stage. Seeking help from histopathology and employing a more radical approach at the earliest may prevent recurrence or morbidity at a later stage.

The aim of our study was to observe the epidemiology and clinic pathological findings in patients with nasal and paranasal masses presenting to our institute and discuss the challenges faced in proper diagnosis and management due to similar presentations, and the role of HPE and immunohistochemistry (IHC) to overcome these challenges.

## Materials and methods

This retrospective observational study was conducted in the Department of Otorhinolaryngology and Department of Pathology of a tertiary care center in North India and involved samples received for histopathological examination over a period of four years, i.e., from September 2018 to August 2022. The study was conducted after obtaining due approval from the Institutional Ethics committee.

Biopsy samples of all the patients clinically diagnosed as nasal or paranasal lesions were included in this study. The medical records of these patients were accessed and relevant clinical details and investigations were retrieved for tabulation. Routinely, all the specimens were sent in 10% neutral buffered formalin for examination as biopsy for diagnosis or after excision. After adequate fixation, the biopsy specimen was submitted for routine processing, followed by paraffin embedding, and stained with hematoxylin and eosin (H&E). Special stains like periodic acid-Schiff (PAS) and Ziehl-Neelsen (ZN) stains for acid-fast bacilli (AFB) were used wherever necessary. IHC was performed in the required samples. IHC was performed with the Automated Leica Bond Max machine (Leica Biosystems, Wetzlar, Germany) using polymer HRP kits of the same company. IHC markers were performed on representative paraffin-embedded sections according to the streptavidin-biotin immunoperoxidase technique.

The findings were noted, and histology was correlated with clinical presentations and investigations, tabulated, and statistically analyzed using SPSS Statistics (IBM, Armonk, NY).

## Results

A total of 396 cases of sinonasal masses were included in our study. Of these, 67.92%% were non-neoplastic lesions whereas 18.18% came out to be benign neoplasms and 13.88% were malignant lesions on HPE (Table 1).

**Table 1:** Age and sex distribution of patients with nose and paranasal lesions

	Inflammatory/infective		Total	Benign		Total	Malignant		Total	%
Age (years)	Male	Female		Male	Female		Male	Female		
0-20	12	04	16	18	-	18	-	1	01	35 (08.84%)
21-40	56	57	113	18	14	32	04	05	09	154 (38.80%)
41-60	64	40	104	14	4	18	10	10	20	142 (35.85%)
>60	23	13	36	2	2	04	23	2	25	65 (16.41%)
Total	155	114	269	52	20	72	37	18	55	396

Nasal obstruction was the most common presenting symptom (73.23% of patients) followed by nasal mass seen in 64.14% of cases (Table 2).

**Table 2:** Clinical presentations of sinonasal lesions

Presentation	No. of cases	Percentage
Nasal obstruction	290	73.23
Nasal discharge	119	30.05
Nasal mass	254	64.14
Epistaxis	67	16.91
Headache	64	16.16
Hyponasality	143	36.11
External facial deformity	65	16.41

Inflammatory sinonasal polyps were the most prominent cases in our study, accounting for 41.16% of all lesions. The male-to-female ratio was 1:1. Histologically, polypoidal tissue was lined by ciliated pseudostratified columnar epithelial cells while stoma consisted of densely packed inflammatory cells like lymphocytes, plasma cells, and polymorphs along with loosely arranged mucous glands. Few polyps showed predominantly eosinophilic cells pointing to an underlying allergic component of the lesion. This feature may help differentiate between polyps arising due to inflammatory rhinosinusitis and those due to allergy.

Many fungal rhino-sinusoidal lesions (18.68%) were seen in our study, which could be attributed to the fact that the study period spanned the peak time of the coronavirus disease 2019 (COVID-19)-associated mucormycosis epidemic. Histologically, mucormycosis was the most prominent type followed by invasive aspergillosis. Cases of nasolabial cysts accounted for 2.52% of the total and presented with nasal mass and nasal obstruction. On histopathology, the cyst showed ciliated pseudostratified columnar epithelial with stoma containing mixed inflammation (Table 3).

**Table 3:** Distribution of inflammatory/infective lesions of the nose and PNS PNS: paranasal sinus; RS: rhinosinusitis

Lesion	No. of cases by location				TOTAL	No. of cases by age				Total
	Nose		PNS			0-20 years	21-40 years	41-60 years	>60 years	
	M	F	M	F						
Polyps	4	3	80	76	163	16	74	57	16	163 (41.16%)
Rhinophyma	1	1	-	-	02	-	-	2	-	02 (0.54%)
Rhinoscleroma	1	2	-	-	03	-	2	1	-	03 (0.75%)
Rhinosporidiosis	1	2	-	-	03	-	-	2	1	03 (0.75%)
Fungal RS	26	8	30	10	74	-	24	32	18	74 (18.68%)
Tuberculosis	3	2	-	-	05	-	2	2	1	05 (1.3%)
Rhinosinusitis	3	6	-	-	09	-	6	3	-	09 (2.3%)
Nasolabial cyst	6	4	-	-	10	-	5	5	-	10 (2.52%)
Total	45	28	110	86	269	16	113	104	36	269

The majority of benign neoplasms encountered were Schneiderian papilloma or inverted papilloma (06.81%). It had a male-to-female ratio of 3: 1. On HPE examination, these showed hyperplastic squamous epithelial cells growing endophytically. Stoma in these lesions consisted of chronic inflammatory cells. This histology needs closer differential with nasal polyps with squamous metaplasia and invasive carcinoma sometimes.

In our study, there were 4.54% cases of juvenile nasopharyngeal angiofibroma (JNA). They were seen only in males, and the majority of cases were in the age group of 0-20 years. On HPE, variable wall thickness with staghorn blood vessels and stellate cells mixed in loose stoma was seen, and the presence of fibro-collagenous substances and inflammatory cells was observed. Hemangiomas accounted for 02.27% of cases. Swelling in and on the nose was the most prominent symptom followed by a nasal obstruction in these cases. On HPE, these lesions showed vascular proliferation, edema, and thin fibrous septae filled with edema.

Nasal schwannoma was seen in 1.01% of cases. On HPE, it showed Antoni A and B areas. Thick-walled hyalinized blood vessels with Verocay bodies were seen in these lesions (Table 4).

**Table 4:** Distribution of benign neoplasms of the nose and PNS PNS: paranasal sinus

Lesion	No. of cases by location				Total	No. of cases by age				Total
	Nose		PNS			0-20 years	21-40 years	41-60 years	>60 years	
	M	F	M	F						
Sinonasal papilloma	6	4	14	3	27	-	17	8	2	27 (06.81%)
Hemangioma	2	7	-	-	09	-	4	3	2	09 (02.27%)
Angiofibroma	18	-	-	-	18	14	4	-	-	18 (04.54%)
Ossifying fibroma	-	-	-	3	03	2	1	-	-	03 (0.75%)
Adenomatoid hamartoma	3	-	-	-	03	-	-	3	-	03 (0.75%)
Neuroma/schwannoma	2	2	-	-	04	2	2	-	-	04 (1.01%)
Leiomyoma	2	1	-	-	03	-	1	2	-	03 (0.75%)
Ameloblastoma	-	-	4	-	04	-	2	2	-	04 (1.01%)
Pleomorphic adenoma	1	-	-	-	01	-	1	-	-	01 (0.25%)
Total	34	14	18	6	72	18	32	18	4	72

Epithelial malignancies arise from the mucosa, olfactory epithelium, and minor salivary glands of the region. These malignancies present with nasal mass, discharge, and epistaxis followed by facial swelling. Eighteen (4.54%) cases of SCC were seen in our study. SCC is the most common malignant tumor. The majority of cases were seen in the fifth-sixth decade of life with an overall male-to-female ratio of 2:1. Nasal obstruction was the most common symptom followed by facial swelling, epistaxis, and palatal perforation. In our cohort, 2.77% of cases were of adenoid cystic carcinoma. On HPE, SCC showed nests, sheets, and cords of tumor cells with mild to moderate pleomorphism; moreover, intracellular and extracellular keratin was seen along with mitotic figures. Out of 18 cases of SCC, moderately differentiated SCC carcinoma comprised 10/18 cases followed by poorly differentiated SCC (5/18) and nonkeratinizing SCC (3/18). IHC for p40 was performed in all the cases of nonkeratinizing SCC and showed strong and diffuse nuclear positivity (Table 5).

**Table 5:** Distribution of malignant neoplasms of the nose and PNS PNS: paranasal sinus

Lesion	No. of cases by location				Total	No. of cases by age				Total
	Nose		PNS			0-20 years	21-40 years	41-60 years	>60 years	
	M	F	M	F						
Adenoid cystic carcinoma	3	4	2	2	11	-	1	3	7	11 (02.77%)
Squamous cell carcinoma	3	3	9	3	18	-	-	7	11	18 (04.54%)
Sinonasal undifferentiated carcinoma	1	-	3	-	04	-	1	3	-	04 (1.01%)
Lymphoma	1	1	3	-	05	-	-	3	2	05 (01.26%)
Olfactory neuroblastoma	3	-	3	1	07	-	4	2	1	07 (01.76%)
High-grade sarcoma	1	-	2	-	03	-	2	1	-	03 (0.75%)
Malignant melanoma	1	1	-	-	02	-	-	-	2	02 (0.54%)
Non-salivary adenocarcinoma (intestinal type)	1	1	-	-	02	1	1		-	02 (0.54%)
Basal cell carcinoma	1	2	-	-	03	-	-	1	2	03 (0.75%)
Total	15	12	22	06	55	01	09	20	25	55

In our study, 2.77% (n=11) cases were of adenoid cystic carcinoma, which predominantly showed tubular and cribriform patterns with a high nuclear-to-cytoplasmic ratio and with myxoid or hyalinized globules. Four cases of sinonasal undifferentiated carcinoma (SNUC) were also seen. Given the undifferentiated nature of this malignancy, IHC analysis was very helpful. SNUC showed positive staining for CK7. Vimentin, CD56, and P40 were negative, confirming the diagnosis.

Olfactory neuroblastoma was seen in 1.76% (n=7) of cases. These lesions showed lobules and nests of tumor cells with round nuclei and scant cytoplasm in the fibrillary background with brisk mitotic activity. Tumor cells were positive for pan-cytokeratin (Pan CK), synaptophysin, vimentin, and S100 (focal) and negative for CD45, CD99, and desmin. Five (1.26%) cases of non-Hodgkin’s lymphoma (NHL) were reported in our study. On microscopic examination, tumor cells were arranged in sheets and clusters. The individual cells were small to medium in size with opened nuclei and conspicuous nucleoli. Necrosis was also seen in a few places. IHC was positive for CD45 in tumor cells and negative for other panels of markers, confirming the diagnosis of NHL. Out of five cases, two cases were diagnosed as B-cell NHL (positive for CD20). The remaining three cases were of T-cell NHL (CD3-positive) (Table 6).

**Table 6:** IHC features of various tumors IHC: immunohistochemistry; NHL: non-Hodgkin's lymphoma

Histological types		IHC
Adenoid cystic carcinoma		CD117-, S100-, CK7-, p63-, and SMA-positive; GFAP-negative
Squamous cell carcinoma		p40-positive
Sinonasal undifferentiated carcinoma		CK7-positive; vimentin-, CD56-, p40-negative
Lymphoma	B-cell NHL	CD45- and CD20-positive; CD3-negative
	T-cell NHL	CD45- and CD3-positive, CD20-negative
Olfactory neuroblastoma		Pan-CK-, synaptophysin-, vimentin-, and S100-positive; CD45-, CD99-, and desmin-negative
High-grade sarcoma		Vimentin- and SMA-positive; desmin-, HMB45-, and CD34-negative
Malignant melanoma		Melan A- and HMB 45-positive; pan-CK-negative
Embryonal rhabdomyosarcoma		Desmin- and vimentin-positive; pan-CK-focally positive, CD45- and CD99-negative
Schwannoma		S100-positive (diffuse)

Malignant melanoma was seen in 0.54% (n=2) of cases, which showed tumor tissue composed of round to oval cells, having moderate pleomorphism, hyperchromatic nuclei, and prominent nucleoli. Melanin pigments were also noted. The diagnosis was confirmed by IHC S100 and HMB45 antibodies.

## Discussion

**Figure 1:**
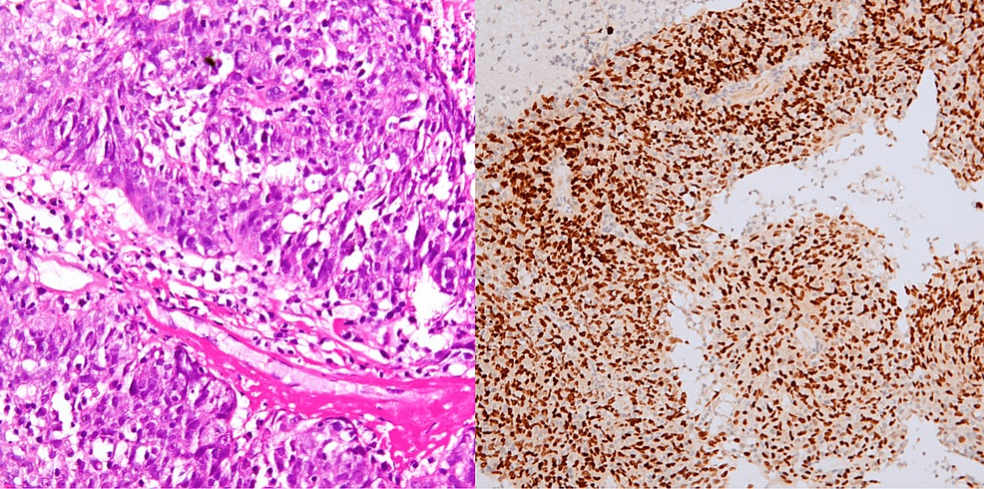
Squamous cell carcinoma Left: Nonkeratinizing squamous cell carcinoma, H&E 200x. Right: Strong and diffuse nuclear positivity for p40, IHC 200x

The nasal cavity is the site of most varied presentations of tumors in the upper respiratory tract [1]. Mass in the nose and PNS form a heterogeneous group of lesions having varied histopathological features. Authors have reported the incidence of sinonasal lesion samples arriving for HPE to be around 16.71%, out of which non-neoplastic lesions accounted for 14.42% and the rest were neoplastic [3] (Figure 1).

In the present study, the age of patients ranged from 12 to 78 years with a mean of 40.34 +3.4 years. Most of the patients (38.80%) were in the age group of 21-40 years, followed by 35.85% of patients in the 41-60 age group. There was a male preponderance in our study with a male-to-female ratio of 1.6:1. In a similar study conducted by Bakari et al., the more prevalent age group was 21-50 years [4]. Also, in a study by Zafar et al., the male-to-female ratio was 1.2:1 [5]. The nose and PNS lesions had similar symptoms of nasal obstruction, nasal discharge, nasal mass, epistaxis, and external nasal deformity in our study and similar studies performed by other authors [1,5,6], but Bakari et al. [4] also reported anosmia as a major symptom, which was not seen in our study.

**Figure 2:**
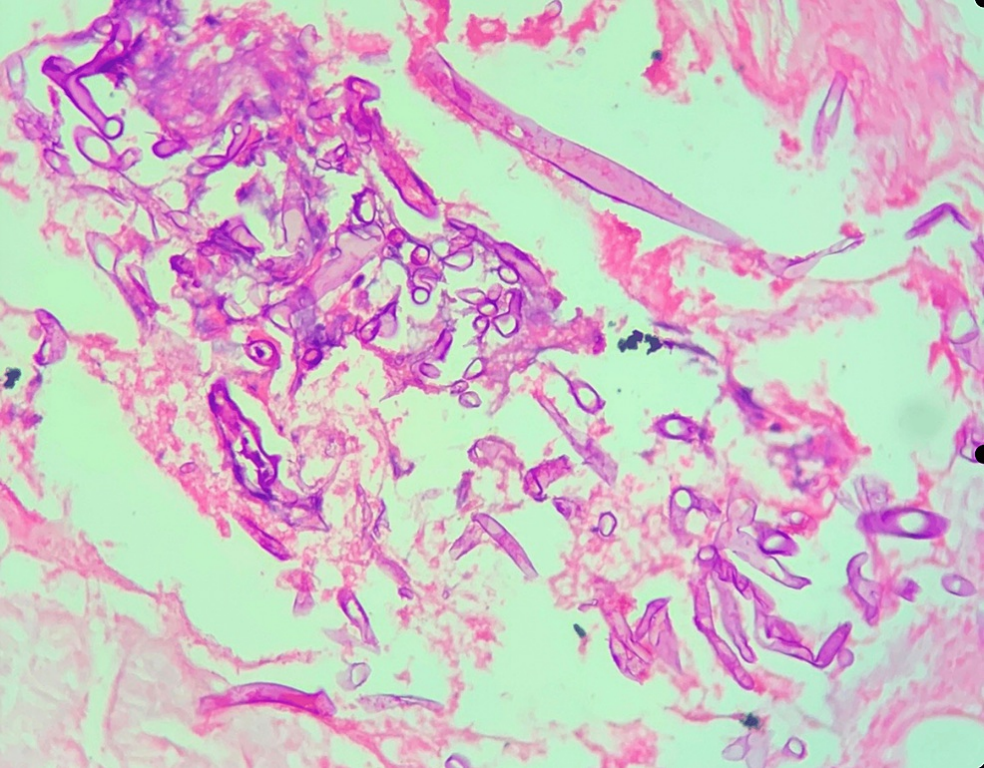
Mucormycosis Large areas of necrosis and hemorrhage with numerous enmeshed fungal elements

Among the non-neoplastic lesions, nasal polyps were the most common ones. In our study, 41.16% were nasal polyps, forming a major part of the non-neoplastic group. Khan et al., in their study [7], reported that 83.33% of their study group had nasal polyps. They also reported 5.55% rhinoscleroma cases, but this was not the case in our study. Such a varied presentation may be due to differences in geographical areas and ethnicities of patients. In our study, there was a high number of fungal RS, especially mucormycosis, and it was due to a sudden surge of mucor cases during the COVID-19 pandemic (Figure 2). Clinically, nasal tumors can easily be misdiagnosed as inflammatory conditions and pose a difficult diagnosis as the presenting features can often be misleading.

**Figure 3:**
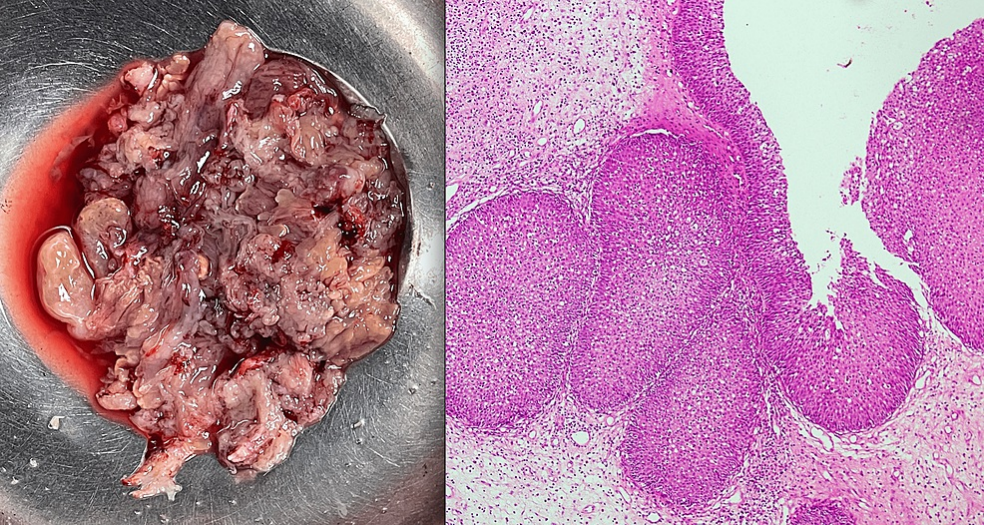
Inverted papilloma Endophytic growth of epithelial nests with smooth contour

Among the benign tumors, non-epithelial tumors were less common than epithelial tumors. Inverted papilloma was the most common epithelial tumor seen in our study and was present in 6.81%, followed by angiofibromas, which were seen in 4.54% of the cohort (Figure 3). Khan et al., in their study of 56 benign tumors, reported a higher incidence of angiofibromas than inverted papillomas. Many studies have been performed on inverted papilloma as these tumors are multi-centric and clinically problematic with a marked tendency for aggressive local involvement; also, recurrence is commonly seen after surgical excision and even transformation to epithelial malignancy (5-9%) [8].

Even though malignant nasal tumors have a very low incidence, they cause a lot of morbidity due to their long course and frequent local recurrences. Nasal tumors tend to become polypoidal. Epithelioid papilloma of the nasal cavity often resembles a nasal polyp. Sinonasal carcinomas were the most common malignant neoplasms in our study; 4.54% of these were SCC and 2.77% were adenoid cystic carcinomas. A similar predominance of SCC was highlighted by Humayun et al. [9] and Raj et al. [10]. Tobacco and air pollution have been implicated in the pathogenesis of SCC and these are very common in this part of the country. Khan et al. have pointed out the high number of nasopharyngeal carcinomas in their study, but such cases were absent in our study [7].

**Figure 4:**
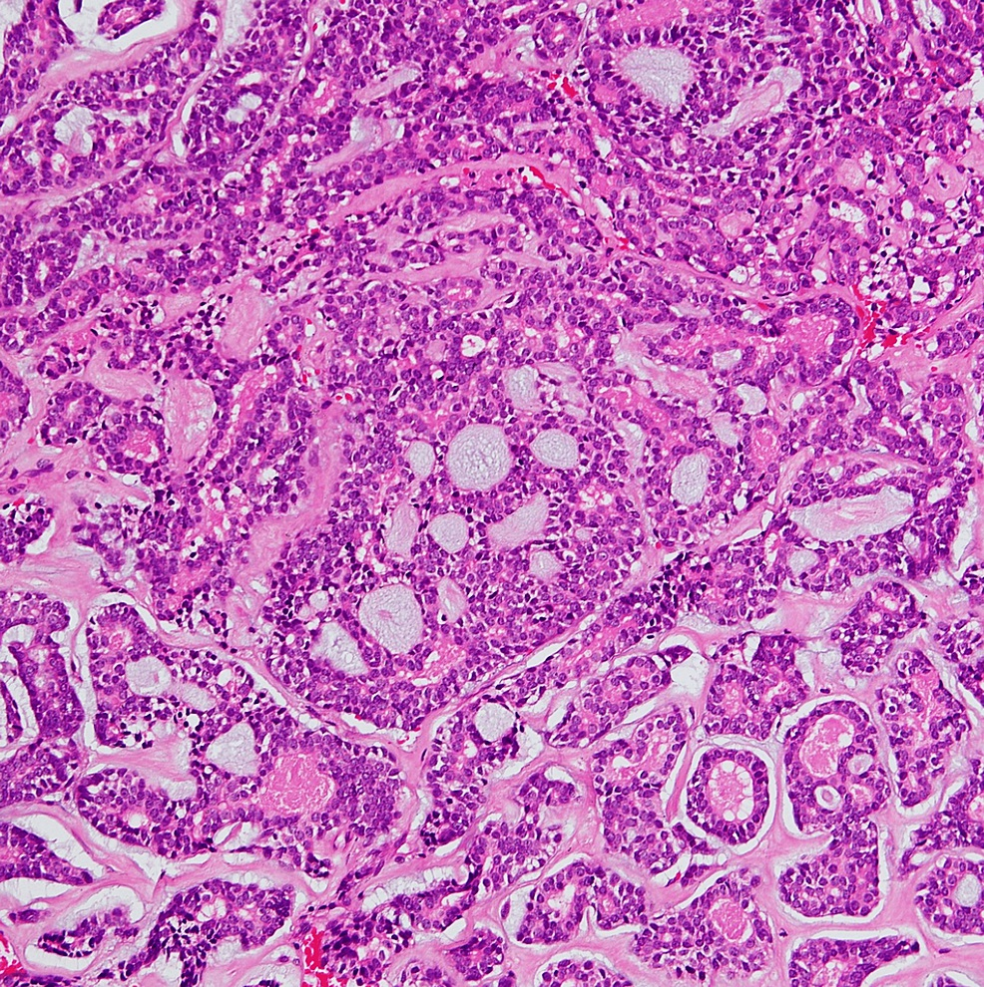
Adenoid cystic carcinoma The cribriform pattern comprised predominantly of myoepithelial cells with myxoid globules, H&E 200x

**Figure 5:**
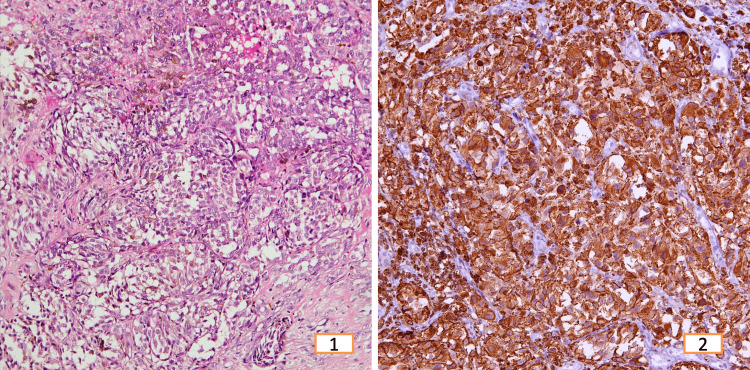
Malignant melanoma (1) The image shows nests of tumor cells with melanin pigment, H&E 200x. (2) Strong cytoplasmic positivity on HMB45 stain, IHC 200x

**Figure 6:**
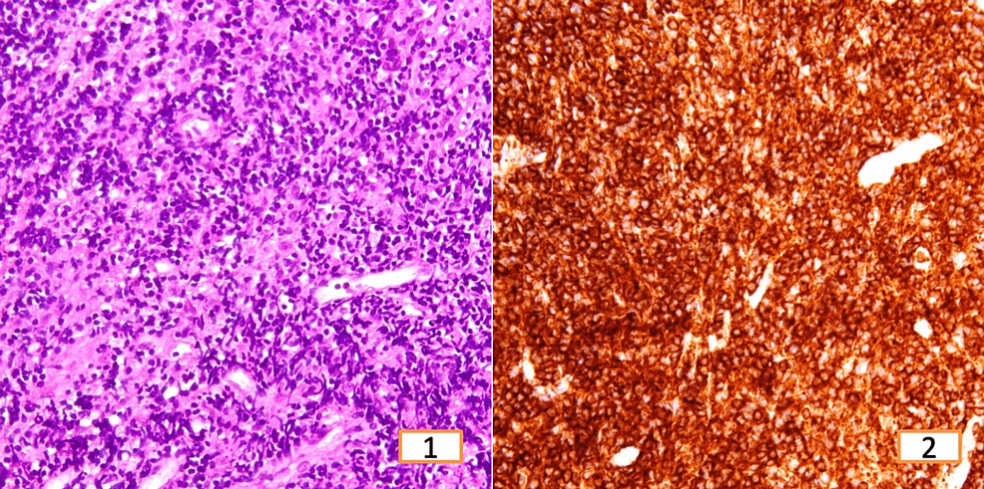
T-cell non-Hodgkin’s lymphoma (1) Small to medium-sized mononuclear cells with oval to irregular nuclei, dispersed chromatin, distinct nucleoli, and scant cytoplasm, H&E 400x. (2) Tumor cells show strong positivity for CD3, IHC 400x

Adenocarcinoma of the sinonasal tract is broadly classified as salivary and non-salivary adenocarcinoma. Non-salivary adenocarcinomas constitute around 10-20% of sinonasal malignant tumors [8]; but in our study, such cases only accounted for 0.50% of cases. Salivary adenocarcinomas arise from surface epithelium. Adenoid cystic carcinoma was seen in 2.77% of our patients (Figure 4). Adenoid cystic carcinomas are more frequently encountered in such lesions and are aggressive tumors [11-14]. Two cases of malignant melanoma of the nose were also seen in our study, clinically presenting with a small mass in the vestibule (Figure 5). Our study also highlighted the presence of lymphoma as an uncommon cause of nasal masses, with 1.26% of cases (Figure 6).

For all the maxillary sinus malignancies, the lateral rhinotomy approach was used for performing maxillectomy. In most cases of proven malignancies, post-surgical radiation is an important part of management. Nasolabial cysts were surgically excised through the sub-labial route. Cases of inverted papillomas were treated surgically by endoscopic medial maxillectomy followed by close follow-up. No cases required revision surgery. All cases of angiofibromas were managed by endoscopic excision using modified Denker’s technique without preoperative embolization with the help of coblation. Hemangiomas were treated by electrocoagulation. Various authors have also advocated the need for prompt surgical management of these lesions [15,16].

## Conclusions

Nose and paranasal masses present as a wide variety of common and rare conditions in clinical practice. It is very important to recognize the spectrum of non-neoplastic and neoplastic lesions in our region. In our study, we concluded that HPE examination is an important differentiating modality in patients who present with similar clinical complaints but varied underlying pathology. We also concluded that the use of IHC is very important in cases with a confusing histopathological picture due to overlapping morphology. IHC should be widely used for distinguishing between closely resembling pathologies. The proximity of the area to the eyes and brain warrants early definitive diagnosis so that the lesion is treated before it can involve important and vital centers.

Clinical diagnosis can be confusing due to similar presentations and appearances, and hence histological examination is an important tool for the timely diagnosis and better management of such patients.
